# Functional analyses of rare germline* BRCA1* variants by transcriptional activation and homologous recombination repair assays

**DOI:** 10.1186/s12885-023-10790-w

**Published:** 2023-04-21

**Authors:** Nicola Bassi, Henrikke Nilsen Hovland, Kashif Rasheed, Elisabeth Jarhelle, Nikara Pedersen, Eunice Kabanyana Mchaina, Sara Marie Engelsvold Bakkan, Nina Iversen, Hildegunn Høberg-Vetti, Bjørn Ivar Haukanes, Per Morten Knappskog, Ingvild Aukrust, Elisabet Ognedal, Marijke Van Ghelue

**Affiliations:** 1 Department of Medical Genetics, Division of Child and Adolescent Health, University Hospital of North Norway, Tromsø, Norway; 2 Familial Cancer Center, Haukeland University Hospital, Bergen, Norway; 3 Department of Medical Genetics, Haukeland University Hospital, Bergen, Norway; 4 Department of Clinical Science, University of Bergen, Bergen, Norway; 5 Department of Medical Biology, University of Tromsø, Tromsø, Norway; 6 Present address: Institute for Clinical and Molecular Medicine, Norwegian University of Science and Technology, NTNU, Trondheim, Norway; 7 Northern Norway Family Cancer Center, University Hospital of North Norway, Tromsø, Norway; 8 Department of Biological Sciences, University of Bergen, Bergen, Norway; 9 Department of Medical Genetics, Oslo University Hospital, Oslo, Norway; 10 Department of Clinical Medicine, University of Tromsø, Tromsø, Norway

**Keywords:** Hereditary breast and ovarian cancer, BRCA1, Functional assay, Homologous recombination repair, Transcriptional activation

## Abstract

**Background:**

Damaging alterations in the *BRCA1* gene have been extensively described as one of the main causes of hereditary breast and ovarian cancer (HBOC). *BRCA1* alterations can lead to impaired homologous recombination repair (HRR) of double-stranded DNA breaks, a process which involves the RING, BRCT and coiled-coil domains of the BRCA1 protein. In addition, the BRCA1 protein is involved in transcriptional activation (TA) of several genes through its C-terminal BRCT domain.

**Methods:**

In this study, we have investigated the effect on HRR and TA of 11 rare *BRCA1* missense variants classified as variants of uncertain clinical significance (VUS), located within or in close proximity to the BRCT domain, with the aim of generating additional knowledge to guide the correct classification of these variants. The variants were selected from our previous study “*BRCA1* Norway”, which is a collection of all *BRCA1* variants detected at the four medical genetic departments in Norway.

**Results:**

All variants, except one, showed a significantly reduced HRR activity compared to the wild type (WT) protein. Two of the variants (p.Ala1708Val and p.Trp1718Ser) also exhibited low TA activity similar to the pathogenic controls. The variant p.Trp1718Ser could be reclassified to likely pathogenic. However, for ten of the variants, the total strength of pathogenic evidence was not sufficient for reclassification according to the CanVIG-UK *BRCA1/BRCA2* gene*-*specific guidelines for variant interpretation.

**Conclusions:**

When including the newly achieved functional evidence with other available information, one VUS was reclassified to likely pathogenic. Eight of the investigated variants affected only one of the assessed activities of BRCA1, highlighting the importance of comparing results obtained from several functional assays to better understand the consequences of *BRCA1* variants on protein function. This is especially important for multifunctional proteins such as BRCA1.

**Supplementary Information:**

The online version contains supplementary material available at 10.1186/s12885-023-10790-w.

## Background

Breast cancer is among the most common cancer types and the leading cause of cancer death in women worldwide [1–4]. While most cancer cases are sporadic, an important minority of case are hereditary and caused by disease-causing germline variants in cancer susceptibility genes. Pathogenic variants in *BRCA1* predispose carriers to hereditary breast and ovarian cancer (HBOC), with a lifetime breast and ovarian cancer risk of 65–79% and 36–53%, respectively [2, 3, 5–7].

The *BRCA1* gene is located on chromosome 17q21 and encompasses 23 exons, encoding for a 220 kDa protein consisting of 1863 amino acids (aa) [8]. The protein contains a N-terminal Really Interesting New Gene (RING) domain (aa 22–64), followed by two canonical nuclear localisation signals (NLS) (aa 503–508 and aa 607–614), a coiled-coil domain (aa 1364–1437), and a BRCA1 carboxyl-terminal (BRCT) domain at the C-terminal. The BRCT domain consists of two BRCT repeats (aa 1646–1736 and aa 1760–1855) connected by a linker region [8].

The BRCA1 protein plays a pivotal role in several key cellular processes, such as transcriptional regulation, cell cycle checkpoint activation, and repair of double-stranded DNA breaks by homologous recombination repair (HRR) [9, 10]. The two most studied domains in the BRCA1 protein are the RING and BRCT domains, and the majority of *BRCA1* variants known to cause HBOC are located within these regions [11, 12]. Through interaction with multiple proteins, the RING, BRCT and coiled-coil domains of BRCA1 are involved in the DNA damage response by HRR, which is an important mechanism to prevent tumorigenesis [13, 14]. In addition to HRR, the BRCT domain is involved in transcriptional regulation in response to DNA damage through binding and activating the basal transcriptional machinery [15]. Impaired assembly between the BRCT domain and interacting proteins has been shown to be associated with tumour susceptibility [16]. Accordingly, alterations in the BRCT domain can impair both the HRR and the TA activities of BRCA1 [17, 18].

Reduced costs and use of next generation sequencing (NGS) have lowered the threshold for genetic testing. Consequently, an extensive number of new variants in cancer predisposing genes like *BRCA1* are discovered, including variants considered to be of uncertain clinical significance (VUSs) due to limited or conflicting information. Variants classified as VUSs are therefore non-informative with respect to clinical decision making, as the associated cancer risk is not known.

A great majority of the VUSs in *BRCA1* are rare missense variants for which very little is known. When investigating the potential effect of variants on protein function, functional assays can be used as supporting to very strong evidence for pathogenic and benign classification [19]. As the BRCA1 protein is involved in a myriad of cellular processes, multiple assays covering different functions of the protein are highly beneficial. According to the *BRCA1* gene-specific variant interpretation guidelines from CanVIG-UK, five published functional protein studies are suggested with specific recommendations regarding the strength of their respective functional evidence, and among these are the transcriptional activation (TA) and homology-directed recombination repair (HDR) assays [20]. In this study, we have therefore assessed the effect on protein expression, HRR and TA of 11 rare missense VUSs located in the BRCT domain of BRCA1, aiming to generate additional knowledge to guide the correct classification of these variants.

## Materials and methods

### Selection of *BRCA1* variants

The 11 *BRCA1* variants (listed in Tables 1 and 2) analysed in this study were selected from our previous study “*BRCA1* Norway”, which is a collection of all *BRCA1* variants detected in the four diagnostic genetic laboratories in Norway [21]. The variants were classified as VUSs by one or more of the Norwegian medical genetic departments and/or in the ClinVar database at the time of selection.

**Table 1:** Functional analyses of *BRCA1* missense variants by protein expression, HDR and TA assay. The 11 *BRCA1* variants of interest were introduced in both *His*-*BRCA1* and *DBD-BRCT* plasmids. The full-length *His-BRCA1* plasmid was used in the HDR assay, while the fusion *DBD-BRCT* plasmid was used in the TA-assay. Both plasmids were used in protein expression analysis. All assays were performed in triplicates, and the values are expressed as mean (%) ± standard deviation (SD) relative to WT (set to 100%). The table summarises the numeric values corresponding to all the functional assays for the 11 *BRCA1* variants of interest, in addition to benign and pathogenic control variants analysed in all assays

BRCA1 variant		Protein expression analysis		Functional activity assays	
**Nucleotide change**	**Amino acid change**	**Full-length** **His-BRCA1** **(HEK293FT) (%)**	**Fusion protein** **DBD-BRCT** **(HEK293FT)** **(%)**	**HDR assay** **His-BRCA1** **(HeLa-DR-GFP) (%)**	**TA assay** **DBD-BRCT** **(HEK293)** **(%)**
**VUSs**					
c.4315C > T	p.Leu1439Phe	64 ± 12	99 ± 9	19.4 ± 2.9	167 ± 11
c.4603G > A	p.Glu1535Lys	65 ± 12	84 ± 30	19.6 ± 2.4	97 ± 6
c.4884G > A	p.Met1628Ile	48 ± 12	76 ± 6	19.9 ± 1.8	128 ± 13
c.5002 T > C	p.Phe1668Leu	35 ± 13	15 ± 2	19.6 ± 2.3	81 ± 6
c.5101C > A	p.Leu1701Met	47 ± 8	45 ± 10	19.4 ± 2.7	107 ± 9
c.5123C > T	p.Ala1708Val	5 ± 3	3 ± 1	16.0 ± 1.7	16 ± 2
c.5125G > A	p.Gly1709Arg	2 ± 3	7 ± 4	22.4 ± 1.3	43 ± 7
c.5131A > C	p.Lys1711Gln	13 ± 7	12 ± 6	17.8 ± 2.1	42 ± 10
c.5153G > C	p.Trp1718Ser	1 ± 0	1 ± 0	21.4 ± 1.8	2 ± 1
c.5245C > G	p.Pro1749Ala	40 ± 10	23 ± 5	101.3 ± 2.7	88 ± 16
c.5504G > A	p.Arg1835Gln	60 ± 32	59 ± 11	21.4 ± 2.1	108 ± 10
**Benign control variants**					
WT	WT	100 ± 13	100 ± 36	100	100 ± 3
c.4956G > A	p.Met1652Ile	58 ± 15	23 ± 3	92.6 ± 2.9	97 ± 11
c.5411 T > A	p.Val1804Asp	80 ± 26	41 ± 6	99.6 ± 1.8	95 ± 14
**Pathogenic control variants**					
c.5095C > T	p.Arg1699Trp	21 ± 14	3 ± 2	25.1 ± 0.8	16 ± 9
c.5513 T > G	p.Val1838Gly	6 ± 8	1 ± 2	21.9 ± 4.0	1 ± 0

*VUS* Variant of Unknown Significance, *His-BRCA1* Histidine-tagged full-length BRCA1 protein, *HDR* Homology-Directed Recombination repair, *TA* Transcriptional Activation, *DBD-BRCT* DBD fused to the BRCT domain of the BRCA1 protein, *WT* Wild Type

### Plasmids

For the HDR assay, the plasmid pcDNA5-HBT-*BRCA1*-WT, hereafter called *His-BRCA1* WT, was used as full-length template for generation of plasmid variants according to Supplementary Table 1. *His-BRCA1* WT and the negative control variant plasmid pcDNA5-HBT-*BRCA1*-Leu1786Pro were kindly provided by Prof. Jeffrey Parvin (Ohio State University, Columbus, OH, USA) [22]. The fusion plasmid construct GAL4 *DBD*:*BRCA1* (amino acids 1396–1863), hereafter called *DBD-BRCT* WT, used for the TA assay, was kindly provided by Alvaro N. A. Monteiro (H. Lee Moffitt Cancer Center and Research Institute, Tampa, FL, USA). This plasmid expresses the C-terminal part of BRCA1 (amino acids 1396–1863) including the BRCT domain fused to the GAL4 DNA Binding Domain (DBD). In addition, the pGAL4-e1b-Luc (firefly luciferase reporter), phRG-TK (*Renilla* luciferase reporter) and pcDNA3.1 (empty vector) were used in the TA assay.

The 11 *BRCA1* variants of interest, as well as benign and pathogenic control variants, were introduced in both *His-BRCA1* WT and *DBD*-*BRCT* WT plasmids by site-directed mutagenesis, using the QuikChange XL Site-Directed Mutagenesis Kit (Agilent). The sequences of the primers (Sigma-Aldrich) are presented in Supplementary table 1. The plasmids were purified using the NucleoBond Xtra Midi Plus Kit for transfection-grade plasmid DNA (MACHEREY–NAGEL) or the QIAfilter Plasmid Maxi Kit (Qiagen), following the manufacturer’s instructions. The presence of the variants, in addition to the whole *BRCA1* insert, were verified by Sanger sequencing. Nomenclature was used according to the Human Genome Variation Society (HGVS) with the *BRCA1* reference sequence NM_007294.3 [23].

### Cell lines

**Fig. 1:**
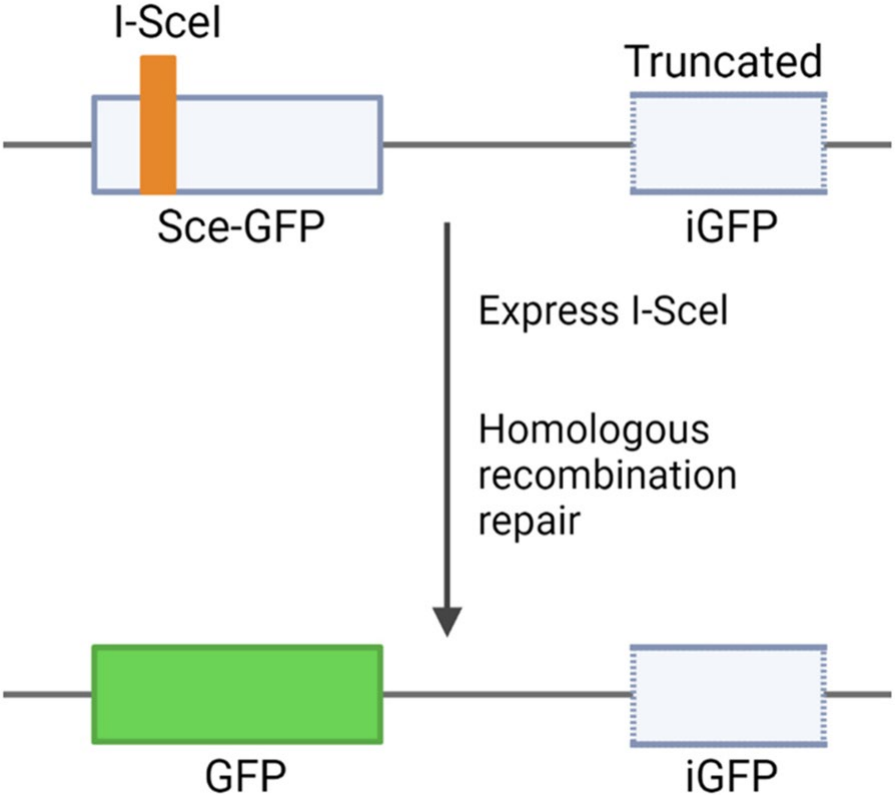
Homology-directed recombination repair (HDR) assay. HeLa-DR-GFP cells are characterised by the presence of two different inactive copies of the gene encoding green fluorescent protein (GFP) integrated in a single locus in the genome. The first copy (Sce-GFP) is inactive due to the presence of a I-SceI cleavage site, and the second copy (iGFP) is truncated at both ends. Transfection of a plasmid encoding the I-Scel endonuclease will result in a double-stranded DNA break of the Sce-GFP copy. During HDR, BRCA1 will use the iGFP copy as a sequence donor to repair the break, and GFP will subsequently be expressed. Figure inspired from [22]

Protein expression levels of the BRCA1 variants (expressed from both *His-BRCA1* and *DBD-BRCT* plasmid constructs) were assessed in HEK293FT cells (Invitrogen). The HRR activity was assessed in HeLa-DR-GFP cells, kindly provided by Prof. Jeffrey Parvin (Ohio State University, Columbus, OH, USA) [22]. These cells are characterised by the presence of two differently inactivated copies of green fluorescent protein (encoded by *GFP*) integrated in a single locus in the genome (Fig. 1). In one of the *GFP* copies, the recognition element for the I-SceI endonuclease has been incorporated. The second *GFP* copy is truncated at both ends. Transfection of HeLa-DR-GFP cells with a plasmid encoding the I-SceI restriction enzyme will cause a double-stranded break in the first *GFP* copy, activating HRR which subsequently utilises the second inactive copy of *GFP* for repair. The repair results in formation of a functional *GFP* gene encoding an active protein, and the recombination can be detected through green fluorescent cells. To evaluate TA activity, HEK293 cells (ATCC) were used. Both HeLa-DR-GFP and HEK293 cells were cultured in Dulbecco’s modified Eagle’s medium (DMEM) supplemented with 10% fetal bovine serum (FBS) (both from Gibco) and 1.5% penicillin–streptomycin (Life Technologies). HEK293FT cells, used to evaluate protein expression levels, were cultured in DMEM high glucose GlutaMAX™ medium (Thermo Fisher Scientific) supplemented with 10% FBS and 1% PenStrep (Sigma-Aldrich).

### Protein expression analysis

Due to the large difference in protein size resulting from the construct expressing the BRCT domain fused to DBD (DBD-BRCT WT, 69 kDa) and the full-length plasmid (His-BRCA1 WT, 221 kDa), two different systems (Invitrogen and BioRad) were used for western blotting to evaluate protein expression of the BRCA1 variants. For both systems, HEK293FT cells were lysed in RIPA buffer (supplemented with cOmplete™, EDTA-free Protease Inhibitor Cocktail, Merck) 48 h post transfection, and centrifuged at 13 000 g for 10 min at 4 °C. Protein concentration of the supernatant was measured using the Pierce BCA protein assay kit (Thermo Fisher Scientific). For western blot analysis of the cell lysates transfected with the *His*-*BRCA1* plasmid, 5 µg total protein was mixed with loading buffer and reducing agent (Invitrogen). After 10 min of denaturation at 70 °C, samples were separated on SDS-PAGE using 3–5% Tris–Acetate SDS-PAGE gels (150 V, 75 min). Subsequently, proteins were transferred to a nitrocellulose membrane (30 V, 60 min). To detect the BRCA1 protein, anti-BRCA1 (sc-6954, Santa Cruz) was used as primary antibody and m-IgGκ BP-HRP (sc-516102, Santa Cruz) as secondary antibody. Anti-β-Actin (sc-47778, Santa Cruz) antibody was used as a loading control. Proteins were visualised using SuperSignal™ West Pico PLUS Chemiluminescent Substrate (Thermo Scientific) and the ChemiDOC™ MP imaging system. The signals were quantified using the ImageLab Software (Biorad, version 6.0), and normalised by dividing the densitometric values from the His-BRCA1 band to the densitometric values of the corresponding actin band. Target protein and loading control signals were assured to be in their linear range. The average relative protein expression of the variants compared to the WT (set to 100%) were calculated.

For cell lysates transfected with the *DBD-BRCT* plasmid, 7 µg protein was mixed with Lammeli Sample Buffer and NuPAGE Sample Reducing Agent (Bio-Rad). After 10 min of denaturation at 70 °C, samples were loaded on Mini-Protean TGX Stain-Free Gels. The gels were run in the Mini-Protean Tetra system at 200 V for 30 min with 1 × Tris/Glycine/SDS running buffer. After activation of the gels using the ChemiDoc MP Imaging system, the proteins were transferred to a membrane using Trans-Blot Turbo RTA Transfer Kit, LV PVDF and the Trans-Blot Turbo transfer system, following the provided protocol for TGX Stain-Free Gels. The following antibodies were used; anti-BRCA1 (sc-6954, Santa Cruz) and secondary m-IgGκ BP-HRP (sc-516102, Santa Cruz). In addition to the secondary antibody, 1 × of Precision Protein StrepTactin-HRP Conjugate was added in the blocking buffer to visualise the ladder. Clarity Western ECL Substrate was used for visualisation of the bands, and blots were imaged using ChemiDoc MP Imaging system. To normalise against the total cell lysate, the normalisation channel of the ImageLab Software was applied. The average relative protein expression of the variants compared to the WT (set to 100%) were then calculated.

### Homology-directed recombination repair assay

The HDR assay was assessed as previously described [22, 24] with some modifications. HeLa-DR-GFP cells (40 000/well) were seeded in 24-well plates and grown in DMEM supplemented with 10% FBS without antibiotics. Cells were co-transfected at 60–70% confluency with the appropriate *BRCA1* plasmids (*Hi*s-*BRCA1* WT or variants) in combination with specific 3’UTR siRNA (GCUCCUCUCACUCUUCAGU) to block endogenous *BRCA1* mRNA, using a JetPrime:DNA ratio of 4:1 (Polyplus Transfection) following the manufacturer’s instructions. Twenty-four hours after transfection, the cells were reseeded into 6-well plates in DMEM supplemented with 10% FBS without antibiotics. After another 24 h, the cells were re-transfected with the appropriate *BRCA1* variant plasmids in combination with siRNA and the plasmid encoding endonuclease (I-SceI). Three days after the second transfection, the presence of GFP cells was confirmed by confocal microscopy. Subsequently, cells were treated with trypsin (Sigma-Aldrich), collected, and resuspended in 1 ml of DMEM supplemented with 10% FBS without antibiotics. In total, 10 000 cells from each well were counted by flow cytometry (FC) using a BD LSRFortessa cell analyser (BD Biosciences), and the proportion (%) of GFP-positive (GFP^+^) cells was calculated. All *BRCA1* variants were tested in triplicate in three independent experiments. The results were expressed as mean percentage of GFP^+^ cells ± standard deviation (SD) of the nine obtained values relative to the WT (set to 100%). As experimental controls, the following samples were included in each independent experiment: 1) untransfected cells to evaluate the background fluorescence from the HeLa-DR-GFP cells, 2) cells transfected exclusively with I-Scel and siRNA to ensure that there is no contribution from remaining endogenous BRCA1, 3) cells transfected with I-Scel, siRNA and empty vector (EV) as a vector control, 4) cells transfected with I-Scel and scrambled siRNA (sc siRNA) as control for the WT *BRCA1* specific 3’UTR siRNA, and 5) cells transfected exclusively with I-Scel as control for the maximum expected value of GFP^+^ cells from endogenous BRCA1. Both *BRCA1* specific 3’UTR and scrambled siRNAs were purchased from Eurofins.

### Transcriptional activation assay

A luciferase reporter assay was performed to measure TA activity of the DBD-BRCT WT and variants using the Dual-Luciferase Reporter Assay System from Promega. HEK293 cells were seeded in 24-well plates and co-transfected with the plasmid encoding the fusion protein *DBD-BRCT* WT/variants (0.36 μg), and the two reporter plasmids encoding the firefly luciferase (pGAL4-e1b-Luc, 0.36 μg) and *Renilla* luciferase (phRG-TK, 36 ng) using a JetPrime:DNA ratio of 2:1. After 24 h, cells were washed in cold PBS and lysed in 100 μl passive lysis buffer (Promega). The lysates were incubated on a rocking platform for 15 min, before clearing by centrifugation at 13 000 rpm for 10 min at 4 °C. The luciferase reporter assay was then performed in a white 96-well plate following the manufacturer’s instructions. Before measuring the luminescence signals, the dual injectors were washed 10 times with ultrapure water and primed with Luciferase Assay Reagent II and Stop & Glo reagent. The luminometer injection volume (Microplate luminometer, Centro XS3 LB 960, Berthold) was 100 μl as per manufacturer guidelines, and the injection speed was set to medium. The measured raw signals were normalised by conversion into firefly/*Renilla* ratios. The TA assay was performed in three biological replicates in independent transfections set-ups. Within a given biological replicate, each variant’s luminescence signal was measured in three technical replicates. The mean WT ratio was calculated for each biological replicate, and the mean percentage of TA activity for each variant relative to WT (set to 100%) and standard deviations were calculated.

### Assessment of variant classifications

The Alamut Software (Version 2.15, SOPHiA GENETICS) and the Human Gene Mutation Database (HGMD) professional 2022.1 (QIAGEN) was used for gathering information on the *BRCA1* variants. Reinterpretation of the variants was performed based on new knowledge using the *BRCA1/BRCA2* gene-specific criteria by CanVIG-UK [19, 20]. If there were evidence elements for both pathogenicity and benignity when combining the total CanVIG-UK criteria for a variant, the recommendations from Garrett et al. were applied [25].

## Results

### Assessment of BRCA1 protein expression by western blotting

**Fig. 2:**
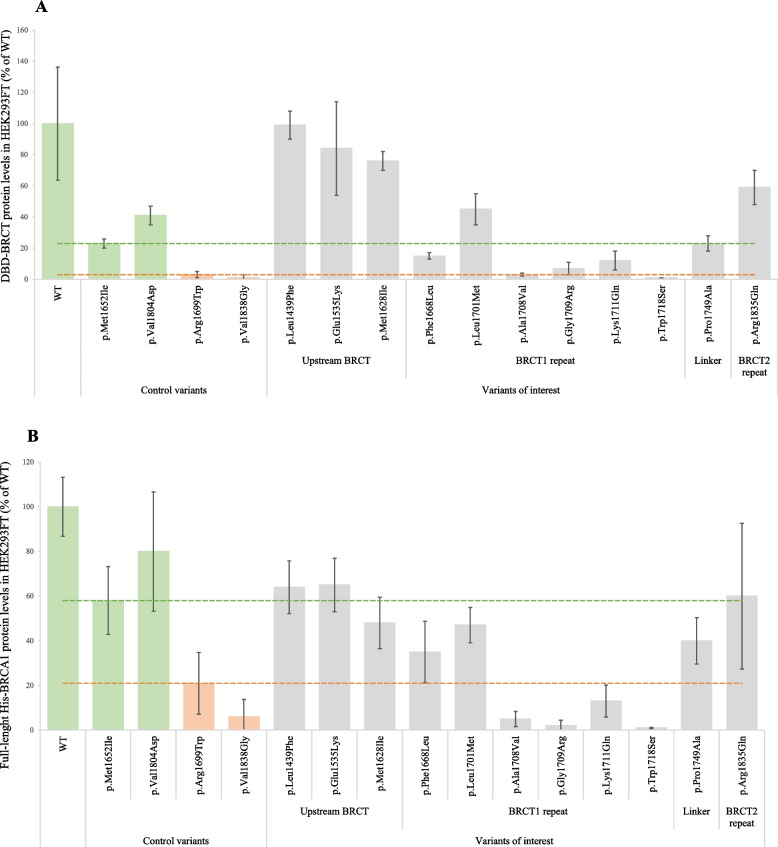
Protein expression levels of the BRCA1 variants as fusion protein (DBD-BRCT) and full-length BRCA1 protein (His-BRCA1). **A** HEK293FT cells were transiently transfected with the plasmid *DBD-BRCT*, known benign and pathogenic control variants, as well as the 11 missense *BRCA1* variants of interest. Western blot bands from 3 biological replicates were quantified in Image Lab software. The BRCA1 bands were normalised against the total protein of each lane. The values are expressed as % relative to the WT (100%). Error bars represent SD. The benign (green) and pathogenic control variants (orange) are grouped to the left. Dashed lines represent estimated thresholds that indicate reduced protein expression levels similar to the pathogenic (below orange line) or benign control variants (above green line). **B** Similar experiment as in A performed for *His-BRCA1* encoding the full-length WT. Actin was used as loading control for normalisation. Footnotes: WT, Wild Type; His-BRCA1, histidine-flagged full-length BRCA1 protein; DBD-BRCT, DNA Binding Domain fused to the BRCT domain of the BRCA1 protein

HEK293FT cells were transfected with the *His-BRCA1* WT/variants and *DBD-BRCT* WT/variant plasmids followed by western blotting. For both the full-length protein and the fusion protein variants, bands corresponding to the expected size for His-BRCA1 (221 kDa) and DBD-BRCT (69 kDa) were detected (Supplementary Figs. 1 and 4). After quantification, the expression levels of BRCA1 protein variants relative to the respective WT protein were calculated. As shown in Fig. 2A (DBD-BRCT) and 2B (His-BRCA1), the two variants p.Ala1708Val and p.Trp1718Ser were found to be expressed at levels similar to the pathogenic controls for both the fusion protein DBD-BRCT and the full-length BRCA1. The variant p.Phe1668Leu was expressed at intermediate levels, i.e. levels between the benign and pathogenic control variants, from both plasmid constructs. The six variants p.Leu1439Phe, p.Glu1535Lys, p.Met1628Ile, p.Leu1701Met, p.Pro1749Ala and p.Arg1835Gln were expressed at levels similar to the WT and/or benign controls in both DBD-BRCT and full-length BRCA1. The two variants p.Gly1709Arg and p.Lys1711Gln were expressed at intermediate protein levels in DBD-BRCT, but at levels similar to the pathogenic controls in His-BRCA1.

### Homology-directed recombination repair assay

**Fig. 3:**
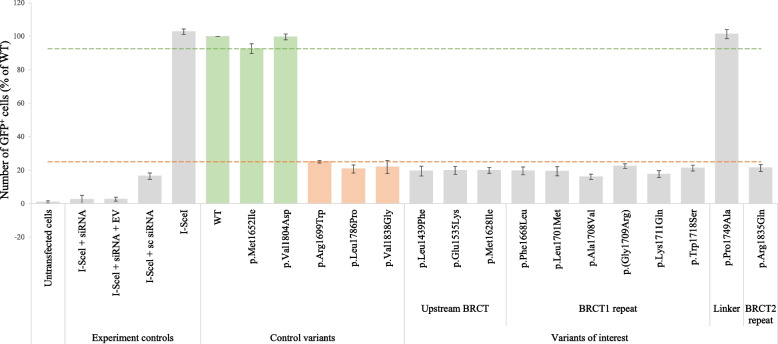
Homologous recombination repair (HRR) activity of BRCA1 protein variants. HeLa-DR-GFP cells were transiently co-transfected with *His*-*BRCA1* WT or variants, together with the plasmid encoding I-Scel endonuclease and siRNA. Known benign (green) and pathogenic (orange) *BRCA1* control variants were included for comparison. As experimental controls (grouped to the left) the following samples were included: untransfected cells to evaluate the background fluorescence from the HeLa-DR-GFP cells, cells transfected exclusively with I-Scel and siRNA to ensure that there is no contribution from remaining endogenous BRCA1, cells transfected with I-Scel, siRNA and empty vector (EV) as a vector control, cells transfected with I-Scel and scrambled siRNA (sc siRNA) as control for the WT BRCA1 specific 3’UTR siRNA, and cells transfected exclusively with I-Scel as control for the maximum expected value of GFP + cells from endogenous BRCA1. Activity was measured in triplicates and in three biological replicates. The mean HRR activities (mean % of GFP^+^ cells) relative to the WT (100%) are shown with error bars depicting the SD. Dashed lines represent estimated thresholds that indicate reduced HRR activity levels similar to the pathogenic (below orange line) or benign control variants (above green line). Footnotes: GFP^+^, Green Fluorescent Protein positive cells; HRR, Homologous Recombination Repair; WT, Wild Type; EV, Empty Vector; I-SceI, endonuclease causing double-stranded break; sc siRNA, scrambled siRNA

The HRR activity was assessed for the full-length WT protein (His-BRCA1 WT), EV, benign and pathogenic control variants, the variants of interest, and a selection of experimental controls by performing HDR assay as described in the methods. As expected, siRNA transfected in combination with I-Scel completely abolished endogenous HRR (Fig. 3, column 2) and showed only a few GFP^+^ cells similar to untransfected cells (Fig. 3, column 1), which has no HRR activation due to lack of I-Scel. This implicates that the endogenous *BRCA1* was completely silenced, and that only the effects of the BRCA1 protein variants expressed from the plasmids were observed in all other samples. The cells co-transfected with I-SceI and scrambled siRNA showed a HRR activity of approximately 20% (Fig. 3, column 4) compared to the cells co-transfected with I-SceI, siRNA and WT *BRCA1* (Fig. 3, column 6). The reduced number of GFP^+^ cells is due to differences in the amount of endogenous BRCA1 protein already present in the cells, compared to the much higher expression of the transiently transfected BRCA1 WT protein. The scrambled siRNA is expected not to affect the endogenous *BRCA1,* and one would expect a number of GFP^+^ cells comparable to that of cells transfected exclusively with I-Scel endonuclease (Fig. 3, column 5). However, our results show that the co-transfection of I-Scel and scrambled siRNA (Fig. 3, column 4) might impair the transfection efficiency of the endonuclease. This would however not affect the results for the variants of interest, as these are co-transfected with the same additional plasmid constructs as the WT (same total DNA input). The raw data output from flow cytometry is reported in Supplementary Fig. 2. Only one variant (p.Pro1749Ala) exhibited HRR activity comparable to the WT BRCA1 (101% of WT activity) and the benign control variants (Fig. 3). All other variants displayed HRR activity from 16–22% of the WT, similarly to the pathogenic control variants which showed HHR activity of 22–25% of the WT. Fluorescence microscopy analyses confirmed these results (Supplementary Fig. 3). Taken together, these data demonstrate that all but one of the variants impaired the BRCA1 HRR activity.

### Transcriptional activation assay

**Fig. 4:**
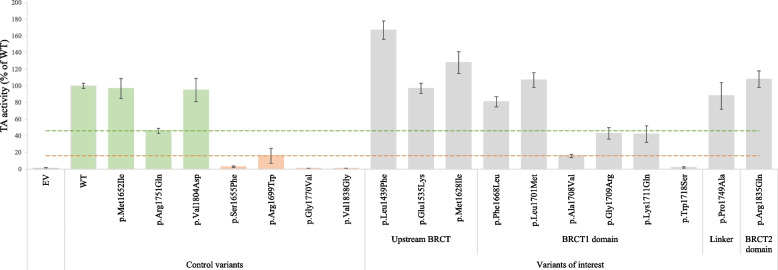
Transcriptional activation (TA) activity of DBD-BRCT protein variants. HEK293 cells were harvested 24 h post-transfection with the *DBD-BRCT* WT or variant, pGAL4-e1b-Luc (firefly) and phRG-TK (*Renilla*). Known benign (green) and pathogenic (orange) *BRCA1* control variants were included for comparison. The Dual-Luciferase Reporter system was used to quantify TA activity. Activity was measured in triplicates and in three biological replicates. TA activity was normalised by dividing firefly fluorescence signal by *Renilla* fluorescence signal. The relative TA activity of each investigated variant was expressed as percentage of WT activity (100%). The mean TA activities are shown with error bars depicting the SD. Dashed lines represent estimated thresholds that indicate reduced TA levels similar to the pathogenic (below orange line) or benign control variants (above green line). Footnotes: TA, Transcriptional Activation; WT, Wild Type; EV, Empty Vector

The TA assay was performed in three biological replicates (each consisting of three technical replicates) measuring each variant’s luminescence signal (Fig. 4). As expected, the TA activity of the pathogenic control variants was low compared to the WT protein (1–16%). The benign controls exhibited TA activity in the range from 46–97% compared to DBD-BRCT WT. Two variants (p.Ala1708Val and p.Trp1718Ser) showed TA activity levels similar to the pathogenic controls (16% and 2%, respectively), while the variants p.Gly1709Arg and p.Lys1711Gln displayed reduced TA activity levels of respectively 43% and 42% compared to the WT, but still similar to the benign control p.Arg1751Gln (46%). The remaining seven variants (p.Leu1439Phe, p.Glu1535Lys, p.Met1628Ile, p.Phe1668Leu, p.Leu1701Met, p.Pro1749Ala and p.Arg1835Gln) exhibited TA activities comparable to or higher than WT BRCA1 and/or benign controls ranging from 68 to 167%. Table 1 summarises the numeric values corresponding to all the functional assays.

## Discussion

In this study, we have analysed the effect of 11 rare missense *BRCA1* VUSs located within or in close proximity to the BRCT domain of BRCA1 with respect to protein expression, HRR and TA activity, with the aim of generating additional knowledge to guide the correct classification of these variants.

The two C-terminal BRCT repeats of the BRCT domain of BRCA1 contain conserved hydrophobic/acidic patches, which have been shown to be crucial for both the HRR capacity and TA activity [17, 18]. In addition, several missense variants located in the BRCT domain have previously been shown to destabilise folding of the BRCA1 protein, leading to increased premature protein degradation [26–28]. Accordingly, prior to investigating the effect of the 11 *BRCA1* variants of interest on HRR and TA, we wanted to assess their effect on protein expression levels in both the DBD-BRCT and full-length versions of the BRCA1 protein. Consistent with previous protein expression studies, several variants exhibited lower protein levels compared to the WT protein [29–31]. Notably, for some of the variants, we observed differences in relative protein expression levels between the full-length His-BRCA1 versus the DBD-BRCT fusion protein versions for the same variant. However, for these variants, there were no clear trends regarding which of the protein constructs being more stably expressed.

Among the 11 analysed variants, p.Ala1708Val and p.Trp1718Ser were the only variants found to show both severely reduced HRR and TA activities, as well as reduced protein expression levels (in both full-length and DBD-BRCT fusion protein), indicating a pathogenic effect (supported by high REVEL scores). Noteworthy, previous functional studies of p.Ala1708Val have revealed several conflicting results. In concordance with our study, the p.Ala1708Val variant has been shown to have decreased protein stability and reduced TA activity, and has also been shown to compromise phosphopeptide binding [31, 32]. However, the same variant has also been shown to have protein stability and HRR capacity similar to WT BRCA1, and intermediate TA activity [33, 34]. Furthermore, the variant was reported as functional in a saturation genome editing study [35]. Studies have shown that TA performed in different cell lines displayed different results [36, 37], potentially explaining the different TA results between this and previous studies. A different missense substitution at the same codon p.(Ala1708Glu) has already been established as pathogenic [38], which suggests that the Ala1708 residue is crucial for the function of BRCA1. Due to the partial defective BRCA1 functions, it has however been suggested that p.Ala1708Val may act as a low or moderate disease risk allele [32, 33, 39]. For the p.Trp1718Ser variant, previous functional studies have revealed that this variant altered BRCA1 function in a saturation genome editing assay, but did not influence splicing (the variant affects the first nucleotide of exon 18) [35, 40]. Different missense substitutions at the Trp1718 codon have been shown to be pathogenic [32, 41–44], supporting an important role for this residue for the function of BRCA1.

The variant p.Pro1749Ala was the only one among the analysed variants found to have activity levels similar to WT/benign controls in both the HDR and TA assays, in addition to protein expression levels similar to WT/benign controls in both DBD-BRCT and full-length protein. This variant is located in the linker region between the two BRCT repeats of the BRCT domain, and this might explain why this variant affects neither the TA activity nor the capacity to mediate HRR. Previously, this variant has been shown to be functional in a saturation genome editing assay and a TA assay [29, 35], and our results are in concordance with previous studies done on variants in the linker region [30]. However, Pro1749 is a highly conserved amino acid, and the linker region is included in the PM1 criteria in the CanVIG-UK B*RCA1* gene-specific guidelines, which states that these regions have a low rate of benign missense variation and that the residue is important for the functional and/or structural properties of BRCA1 [20].

The eight remaining variants (p.Leu1439Phe, p.Glu1535Lys, p.Met1628Ile, p.Phe1668Leu, p.Leu1701Met, p.Gly1709Arg, p.Lys1711Gln and p.Arg1835Gln) all showed severely reduced capacity in HRR, but TA activity levels similar to the WT protein. Recruitment of multiple proteins to the sites of DNA damage along with BRCA1 is essential for HRR, and this is achieved through cascades of protein interactions and the formation of the macro-complex. Among others, BARD1 binds to the RING domain of BRCA1, ABRA1 binds to the BRCT domain, and PALB2 binds to the coiled-coil domain of BRCA1 and works as a recruiter of BRCA2 [45–47]. A potential explanation for the observation of reduced HRR activity and normal TA levels for the eight abovementioned variants could therefore be that these variants, directly or indirectly through conformational changes, alter the binding sites that are crucial for HRR, but not for TA. An alternative explanation could be differences in folding between the full-length and the DBD-BRCT fusion protein [48–50]. Hence, the potential structural changes induced by these variants could affect binding sites necessary for only some and not all of the downstream functions of BRCA1. Furthermore, some variants located in the BRCT domain have been shown to cause retainment of the BRCA1 protein in the cytoplasm, hampering its transition to the nucleus [51, 52]. The cytoplasmic accumulation has been shown to be due to a reduced nuclear import, which again prevents the binding to damaged DNA [51, 52]. This might explain why some of our variants resulted in loss of HRR function, even if they showed protein expression levels comparable to the WT protein.

To the best of our knowledge, p.Arg1835Gln and p.Phe1668Leu are the only variants, in addition to p.Ala1708Val, that have previously been analysed in a HDR assay. The variant p.Arg1835Gln was shown to have partial HRR activity (less than 70%) [39], while p.Phe1668Leu was found to be deleterious in concordance with our results [53]. The variants p.Phe1668Leu, p.Gly1709Arg, and p.Lys1711Gln showed both lower protein expression than the WT protein and had significantly reduced HRR capacity, but exhibited TA capacity comparable to WT BRCA1 and/or benign controls. In concordance with previous studies [30], this indicates that even significantly reduced BRCA1 expression levels are sufficient to perform TA at similar levels as the WT protein, and that protein expression levels do not necessarily correlate to the level of protein activity.

Based on the findings in the functional assays of this study, the interpretation and variant classification of the 11 analysed VUSs was re-evaulated (Table 2) using the *BRCA1/BRCA2* gene-specific guideline for variant interpretation from CanVIG-UK [54]. The PS3/BS3 functional criteria was weighted as strong for those variants where the results in this study were in concordance with published data in any of the five studies approved by CanVIG-UK [20, 29, 34, 35, 53, 55]. All variants for which there were conflicts between the findings in this study and data in the functional studies recommended by CanVIG-UK (like for p.Phe1668Leu), the functional evidence criteria was not used. For variants not investigated in any of the functional studies recommended by CanVIG-UK, the functional criteria was used as supportive strength based on the findings in this study.

To conclude, several of the investigated *BRCA1* variants showed altered BRCA1 protein functions, but when combining the newly achieved functional evidence with other available information for the variants from literature and in silico tools, all variants except one (p.Trp1718Ser), were still classified as VUSs (Table 2) according to the CanVIG-UK *BRCA1/BRCA2* gene-specific recommendations. A higher number of benign and pathogenic control variants have to be included in all functional assays to be able to increase the strength of the functional evidence, and potentially reclassify several variants [56]. The reclassification of p.Trp1718Ser to likely pathogenic will allow better diagnostics, and contribute to correct clinical management of both the patient and family members carrying this variant. Furthermore, we have demonstrated that several of the investigated variants affected only one of the assessed activities of BRCA1, and therefore we highlight the importance of combining several different functional assays when assessing the effects of rare *BRCA1* missense variants. This is especially important for large multifunctional proteins like BRCA1, which is involved in a wide range of functions and interactions with other proteins.

**Table 2:** Classification of *BRCA1* variant of interest. The *BRCA1* variants were classified according to the CanVIG-UK *BRCA1/BRCA2* gene-specific recommendations [20]

**Variant**	**Domain**	**Functional studies recommended by CanVIG-UK** ^a^	**Results in functional assays in this study**	**GnomAD MAF % (allele count)** ^b^	**REVEL** ^c^	**CanVIG-UK criteria**	**ClinVar classifications**	**Revised class**
c.4315C > T p.(Leu1439Phe)		Neutral in cisplatin and DR-GFP HDR assays, not clear in olaparib assay [53]	Reduced HRR and normal TA	0.0007760 (3)	0.25	BP1, BP4	LBx1, VUSx3	VUS •
c.4603G > A p.(Glu1535Lys)			Reduced HRR and normal TA	- (0)	0.59	BP1, PM2_mod, PS3_sup	VUSx3	VUS
c.4884G > A p.(Met1628Ile)			Reduced HRR and normal TA	- (0)	0.55	BP1, PM2_mod, PS3_sup	VUSx1	VUS
c.5002 T > C p.(Phe1668Leu)	BRCT1	Neutral in cisplatin and olaparib assay, deleterious in DR-GFP HDR assay [53] Functional in saturation genome editing assay [35]	Reduced HRR and normal TA	- (1)	0.60	PM1_sup, PM2_sup, PS3_sup		VUS
c.5101C > A p.(Leu1701Met)	BRCT1	Functional in saturation genome editing assay [35]	Reduced HRR and normal TA	- (1)	0.56	PM1_sup, PM2_sup	LBx1, VUSx3	VUS
c.5123C > T p.(Ala1708Val)	BRCT1	Neutral in HDR, localisation and phosphopeptide-binding assays, but expressed and purified with low yield from *E.coli* [34] Functional in saturation genome editing assay [35]	Reduced HRR and reduced TA	0.01314 (7)	0.79	BS1, PM1_sup, PM5_sup, PP3	VUSx16	VUS
c.5125G > A p.(Gly1709Arg)	BRCT1	Functional in saturation genome editing assay [35]	Reduced HRR and normal TA	- (0)	0.78	PM1_sup, PM2_mod, PP3	VUSx5	VUS
c.5131A > C p.(Lys1711Gln)	BRCT1	Functional in saturation genome editing assay [35]	Reduced HRR and normal TA	- (0)	0.65	PM1_sup, PM2_mod,	LBx1, VUSx1	VUS
c.5153G > C p.(Trp1718Ser)	BRCT1	Intermediate function (towards LOF) in saturation genome editing assay [35]	Reduced HRR and reduced TA	- (0)	0.91	PM5_sup, PM2_mod, PP3, PS3_strong	VUSx2, LPx2	LP
c.5245C > G p.(Pro1749Ala)	Linker region	Functional in TA assay [29], and functional in saturation genome editing assay [35]	No alterations	- (1)	0.77	PM1_sup, PM2_sup, PP3, BS3_strong	VUSx2	VUS
c.5504G > A p.(Arg1835Gln)	BRCT2	Intermediate function (towards F) in saturation genome editing assay [35]	Reduced HRR and normal TA	0.002316 (7)	0.50	PS3_sup	LBx1, VUSx9	VUS

^a^According to the CanVIG-UK *BRCA1* specific guideline for variant interpretation, five functional protein studies are suggested with specific recommendations regarding the strength of their respective functional evidence [20, 29, 34, 35, 53, 55]. ^b^Minor allele frequency numbers were retrieved from GnomAD (v2.1.1., non-cancer) Popmax Filtering AF (95% confidence) [57]. ^c^REVEL was used to assess in-silico predictions with a benign cut off at or below 0.4 and a pathogenic cut off at or above 0.7 as recommended in the Best Practice Guidelines for Variant Classification by CanVIG-UK [19, 58]. •This variant could theoretically be classified as likely benign (BP1 and BP4 criteria) according to CanVIG-UK [19, 20], but due to conflicting functional evidence, the variant was still classified as VUS

*MAF* Minor Allele Frequency, *HDR* Homology-Directed Recombination repair assay, *HRR* Homologous Recombination Repair, *TA* Transcriptional Activation, *VUS* Variant of Uncertain Significance, *LB* Likely Benign, *LP* Likely Pathogenic, *LOF* Loss Of Function, *F* Functional

## Supplementary Information

**Additional file 1:Supplementary Table 1.** Primer sequences used in site-directed mutagenesis. **Supplementary Figure 1.** Protein expression levels of BRCA1 variants determined by western blot analysis. (A) The figure shows images from one representative replicate for the fusion protein DBD-BRCT. HEK293FT cells were transiently transfected with *DBD-BRCT *WT, known benign (green) and pathogenic (red) control variants, and 11 missense* BRCA1 *VUSs. Cells were harvested 48 h post transfection, and 7 µg total protein retrieved from cell lysate was analysed per lane by western blotting. BRCA1 was detected with anti-BRCA1 antibody. The BRCA1 bands were normalised against the total protein of each lane. The blots were cut in two prior to incubation with anti-BRCA1 and anti-actin, so each blot are divided into two images. (B) The figure shows images from one representative replicate for the full-length His-BRCA1. HEK293FT cells were transiently transfected with *His*-*BRCA1* WT, known benign (green) and pathogenic (red) control variants, and 11 missense* BRCA1 *VUSs. Cells were harvested 48 h post transfection, and 5 µg total protein retrieved from cell lysate was analysed per lane by western blotting. BRCA1 was detected with anti-BRCA1 antibody. Actin was used as loading control to normalise the corresponding BRCA1 bands. **Supplementary Figure 2.** Representative raw data output from flow cytometry. The settings for analyses were: FSC=60, SSC=220, GFP=235. Footnotes: FSC, Forward SCatter; SSC, Side SCatter; GFP, Green Fluorescent Protein; WT, Wild Type; I-SceI, endonuclease causing double strand break; sc siRNA, scrambled siRNA. **Supplementary Figure 3.** Fluorescent microscope analyses of GFP^+^ cells after HRR induction. The cells were evaluated 24 hours after flow cytometry analyses, using a magnification of 10x. Footnotes: GFP^+^, green fluorescent protein positive cells; HRR, homologous recombination repair; WT, wild type; I-SceI, endonuclease causing double strand break; sc siRNA, scrambled siRNA. **Supplementary Figure 4.** Original western blot images for Supplementary figure 1. The images are not cropped or edited. (A) The figure shows original images from four replicates for the fusion protein DBD-BRCT. Loading order replicate 1: WT, EV, p.Leu1439Phe, p.Phe1668Leu. p.Leu1701Met, p.Ala1708Val, p.Gly1709Arg, p.Lys1711Ser, p.Trp1718Ser, p.Pro1749Ala, not relevant, WT, EV, not relevant*, not relevant, p.Arg1835Gln, not relevant, p.Met1652Ile, p.Val1804Asp, not relevant, p.Arg1699Trp, p.Val1838Gly. Loading order replicate 2: WT, EV, p.Leu1439Phe. p.Met1652Ile, not relevant, p.Leu1701Met, p.Arg1699Trp, p.Leu1701Met, p.Ala1708Val, p.Gly1709Arg, WT, p.Lys1711Ser, not relevant, p.Val1804Asp, not relevant, not relevant, p.Arg1835Gln, not relevant, WT, EV, p.Trp1718Ser, p.Pro1749Ser, p.Val1838Gly. Loading order replicate 3: WT, EV, p.Met1652Ile, p.Val1804Asp, p.Leu1439Phe, p.Phe1668Leu, p.Leu1701Met, p.Ala1708Val, p.Gly1709Arg, p.Lys1711Ser, p.Trp1718Ser, WT, EV, p.Ser1655Phe, p.Arg1699Trp, p.Val1838Gly, p.Pro1749Ala, not relevant, not relevant, not relevant, p.Arg1835Gln, not relevant. Loading order replicate 4: WT, p.Glu1535Lys, p.Met1628Ile, WT, p.Glu1535Lys, p.Met1628Ile, WT, p.Glu1535Lys, p.Met1628Ile. BRCA1 was detected with anti-BRCA1 antibody. The BRCA1 bands were normalised against the total protein of each lane. (B) The figure shows images from four replicates for the full-length His-BRCA1. The blots were cut in two prior to incubation with anti-BRCA1 and anti-actin, so each blot are divided into two images. Loading order replicate 1, 2 and 3: WT, p.Leu1439Phe, p.Glu1535Lys, p.Met1628Ile, p.Phe1668Leu, p.Leu1701Met, p.Ala1708Val, p.Gly1709Arg, p.Lys1711Gn, p.Trp1718Ser, WT, not relevant, not relevant, not relevant, p.Pro1749Ala, p.Arg1835Gln, p.Arg1699Trp, p.Val1838Gly, p.Met1652Ile, p.Val1804Asp. Loading order replicate 4: WT, EV, EV, EV. BRCA1 was detected with anti-BRCA1 antibody. Actin was used as loading control to normalise the corresponding BRCA1 bands. *Samples included in the Western Blot, but not relevant in this study.

## Data Availability

The datasets used and analysed during the current study are available from the corresponding author on reasonable request.
