# Supplementary material for: Functional analyses of rare germline BRCA1 variants by transcriptional activation and homologous recombination repair assays
Source: BMC Cancer. 2023 Apr 21;23:368. doi: 10.1186/s12885-023-10790-w (PMC10122298; doi:10.1186/s12885-023-10790-w)
Supplement: Supplementary file 1 — Additional file 1:Supplementary Table 1. Primer sequences used in site-directed mutagenesis. Supplementary Figure 1. Protein expression levels of BRCA1 variants determined by western blot analysis. (A) The figure shows images from one representative replicate for the fusion protein DBD-BRCT. HEK293FT cells were transiently transfected with DBD-BRCT WT, known benign (green) and pathogenic (red) control variants, and 11 missense BRCA1 VUSs. Cells were harvested 48 h post transfection, and 7 µg total protein retrieved from cell lysate was analysed per lane by western blotting. BRCA1 was detected with anti-BRCA1 antibody. The BRCA1 bands were normalised against the total protein of each lane. The blots were cut in two prior to incubation with anti-BRCA1 and anti-actin, so each blot are divided into two images. (B) The figure shows images from one representative replicate for the full-length His-BRCA1. HEK293FT cells were transiently transfected with His-BRCA1 WT, known benign (green) and pathogenic (red) control variants, and 11 missense BRCA1 VUSs. Cells were harvested 48 h post transfection, and 5 µg total protein retrieved from cell lysate was analysed per lane by western blotting. BRCA1 was detected with anti-BRCA1 antibody. Actin was used as loading control to normalise the corresponding BRCA1 bands. Supplementary Figure 2. Representative raw data output from flow cytometry. The settings for analyses were: FSC=60, SSC=220, GFP=235. Footnotes: FSC, Forward SCatter; SSC, Side SCatter; GFP, Green Fluorescent Protein; WT, Wild Type; I-SceI, endonuclease causing double strand break; sc siRNA, scrambled siRNA. Supplementary Figure 3. Fluorescent microscope analyses of GFP+ cells after HRR induction. The cells were evaluated 24 hours after flow cytometry analyses, using a magnification of 10x. Footnotes: GFP+, green fluorescent protein positive cells; HRR, homologous recombination repair; WT, wild type; I-SceI, endonuclease causing double strand break; sc siRNA, scrambled [file 12885_2023_10790_MOESM1_ESM.docx]

**SUPPLEMENTARY**

**Supplementary table 1:** Primer sequences used in site-directed mutagenesis.

**BRCT Variants aa Primers Sequences Bases**

**repeat**

Upstream c.4315C>T p.(Leu1439Phe) F 5'-ATTTCGCAGGTCCTCAAAGGCAGAAGAGTCACTTA-3' 35

R 5'-TAAGTGACTCTTCTGCCTTTGAGGACCTGCGAAAT-3' 35

Upstream c.4603G>A p.(Glu1535Lys) F 5'-CTTCCAGCTGTTGCTCCTTCACATCAACAACCTTAAT-3' 37

R 5'-ATTAAGGTTGTTGATGTGAAGGAGCAACAGCTGGAAG-3' 37

Upstream c.4884G>A p.(Met1628Ile) F 5'-CTGCTGGGTATAATGCAATAGAAGAAAGTGTGAGCAG-3' 37

R 5'-CTGCTCACACTTTCTTCTATTGCATTATACCCAGCAG-3' 37

1 c.4956G>A p.(Met1652Ile) F 5'-CAGGCCAGACACCACTATGGACATTCTTTTGTTGACC-3' 37

R 5'-GGTCAACAAAAGAATGTCCATAGTGGTGTCTGGCCTG-3' 37

1 c.5002T>C p.(Phe1668Leu) F 5'-AATTTATGCTCGTGTACAAGCTTGCCAGAAAACACCACATC-3' 41

R 5'-GATGTGGTGTTTTCTGGCAAGCTTGTACACGAGCATAAATT-3' 41

1 c.5095C>T p.(Arg1699Trp) F 5'-CTAGAAAATATTTCAGTGTCCATTCACACACAAACTCAGCATCTG-3' 45

R 5'-CAGATGCTGAGTTTGTGTGTGAATGGACACTGAAATATTTTCTAG-3' 45

1 c.5101C>A p.(Leu1701Met) F 5'-CCTAGAAAATATTTCATTGTCCGTTCACACACAAACTCAGCAT-3' 43

R 5'-ATGCTGAGTTTGTGTGTGAACGGACAATGAAATATTTTCTAGG-3' 43

1 c.5123C>T p.(Ala1708Val) F 5'-CGGACACTGAAATATTTTCTAGGAATTGTGGGAGGAAAATGGGTA-3' 45

R 5'-TACCCATTTTCCTCCCACAATTCCTAGAAAATATTTCAGTGTCCG-3' 45

1 c.5125G>A p.(Gly1709Arg) F 5'-CGGACACTGAAATATTTTCTAGGAATTGCGAGAGGAAAATGGGTA-3' 45

R 5'-TACCCATTTTCCTCTCGCAATTCCTAGAAAATATTTCAGTGTCCG-3' 45

1 c.5131A>C p.(Lys1711Gln) F 5'-TTTTCTAGGAATTGCGGGAGGACAATGGGTAGTTAGCTATTTCTG-3' 45

R 5'-CAGAAATAGCTAACTACCCATTGTCCTCCCGCAATTCCTAGAAAA-3' 45

1 c.5153G>C p.(Trp1718Ser) F 5'-GGGAGGAAAATGGGTAGTTAGCTATTTCTCGGTGACCCAGTCTAT-3' 45

R 5'-ATAGACTGGGTCACCGAGAAATAGCTAACTACCCATTTTCCTCCC-3' 45

Linker c.5245C>G p.(Pro1749Ala) F 5'-TTCTCTTGCTCGCTTTGCACCTTGGTGGTTTCTTC-3' 35

R 5'-GAAGAAACCACCAAGGTGCAAAGCGAGCAAGAGAA-3' 35

2 c.5411T>A p.(Val1804Asp) F 5'-CCACAATTGGGTGGTCACCTGTGCCAAGG-3' 29

R 5'-CCTTGGCACAGGTGACCACCCAATTGTGG-3' 29

2 c.5504G>A p.(Arg1835Gln) F 5'-GAGGCACCTGTGGTGACCCAAGAGTGGGTGTTGGACAGT-3' 39

R 5'-ACTGTCCAACACCCACTCTTGGGTCACCACAGGTGCCTC-3' 39

2 c.5513T>G p.(Val1838Gly) F 5'-CCTGTGGTGACCCGAGAGTGGGGGTTGGACAGTGTAGCACTCTAC-3' 45

R 5'-GTAGAGTGCTACACTGTCAAACCCCCACTCTCGGGTCACCACAGG-3' 45

BRCT domain = BRCA1 carboxy-terminal repeats; aa = amino acid; F = forward; R = reverse.

**SUPPLEMENTARY FIGURES:**

**A**


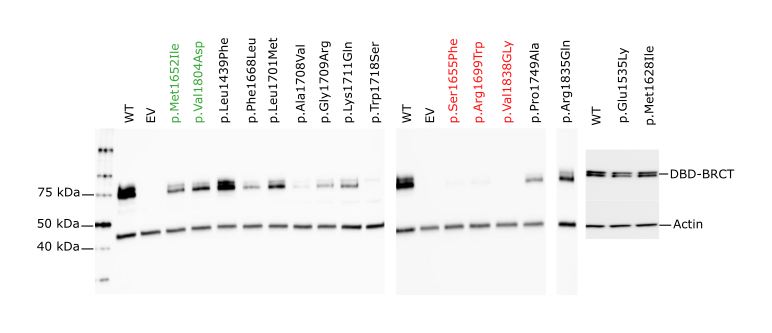


**B**


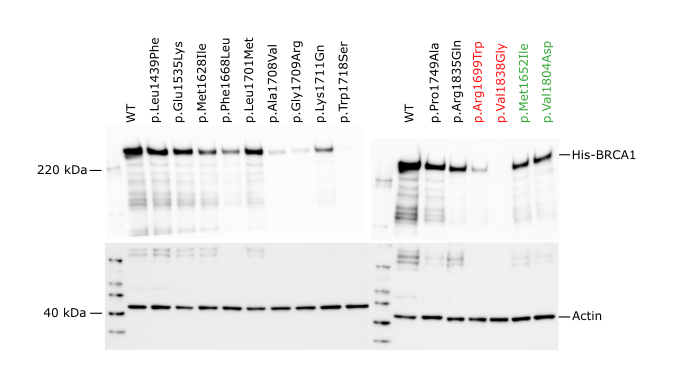


**Supplementary figure 1:** **Protein expression levels of BRCA1 variants determined by western blot analysis. (A)** The figure shows images from one representative replicate for the fusion protein DBD-BRCT. HEK293FT cells were transiently transfected with *DBD-BRCT* WT, known benign (green) and pathogenic (red) control variants, and 11 missense *BRCA1* VUSs. Cells were harvested 48 h post transfection, and 7 µg total protein retrieved from cell lysate was analysed per lane by western blotting. BRCA1 was detected with anti-BRCA1 antibody. The BRCA1 bands were normalised against the total protein of each lane. The blots were cut in two prior to incubation with anti-BRCA1 and anti-actin, so each blot are divided into two images.  **(B)** The figure shows images from one representative replicate for the full-length His-BRCA1. HEK293FT cells were transiently transfected with *His*-*BRCA1* WT, known benign (green) and pathogenic (red) control variants, and 11 missense *BRCA1* VUSs. Cells were harvested 48 h post transfection, and 5 µg total protein retrieved from cell lysate was analysed per lane by western blotting. BRCA1 was detected with anti-BRCA1 antibody. Actin was used as loading control to normalise the corresponding BRCA1 bands.

**
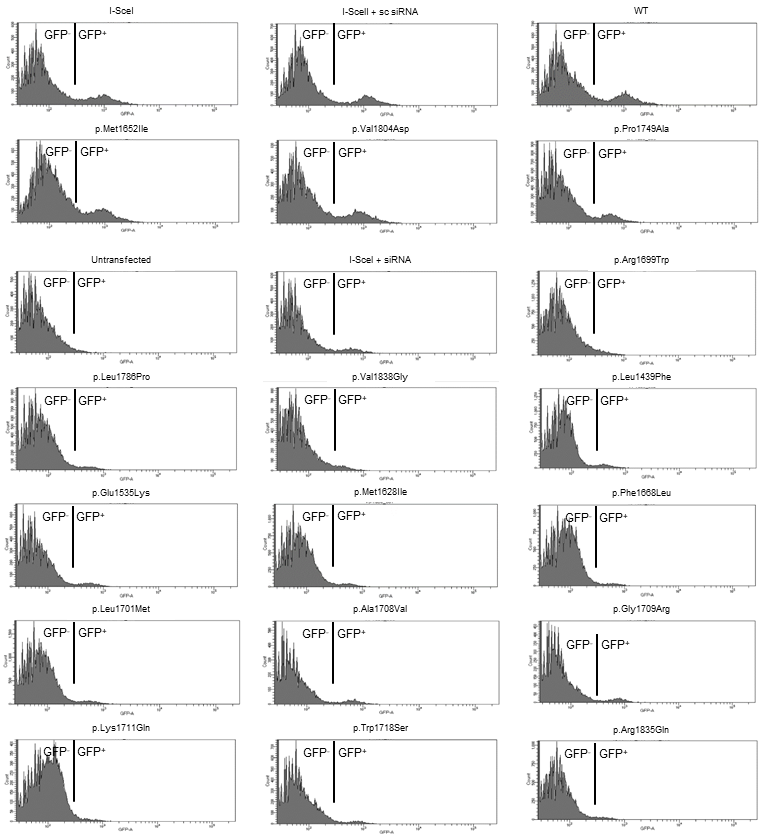
**

**Supplementary figure 2: Representative raw data output from flow cytometry.** The settings for analyses were: FSC=60, SSC=220, GFP=235.

**Footnotes:** FSC, Forward SCatter; SSC, Side SCatter; GFP, Green Fluorescent Protein; WT, Wild Type; I-SceI, endonuclease causing double strand break; sc siRNA, scrambled siRNA.

**
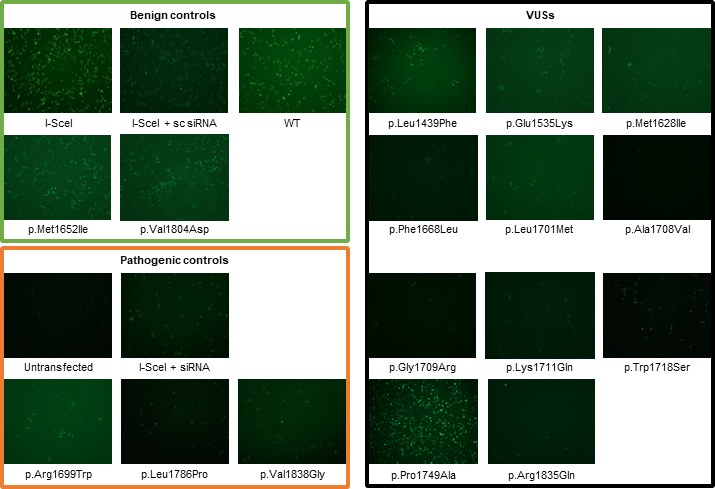
**

**Supplementary figure 3: Fluorescent microscope analyses of GFP^+^ cells after HRR induction**. The cells were evaluated 24 hours after flow cytometry analyses, using a magnification of 10x.

**Footnotes:** GFP^+^, green fluorescent protein positive cells; HRR, homologous recombination repair; WT, wild type; I-SceI, endonuclease causing double strand break; sc siRNA, scrambled siRNA.

**A**

Replicate 1.


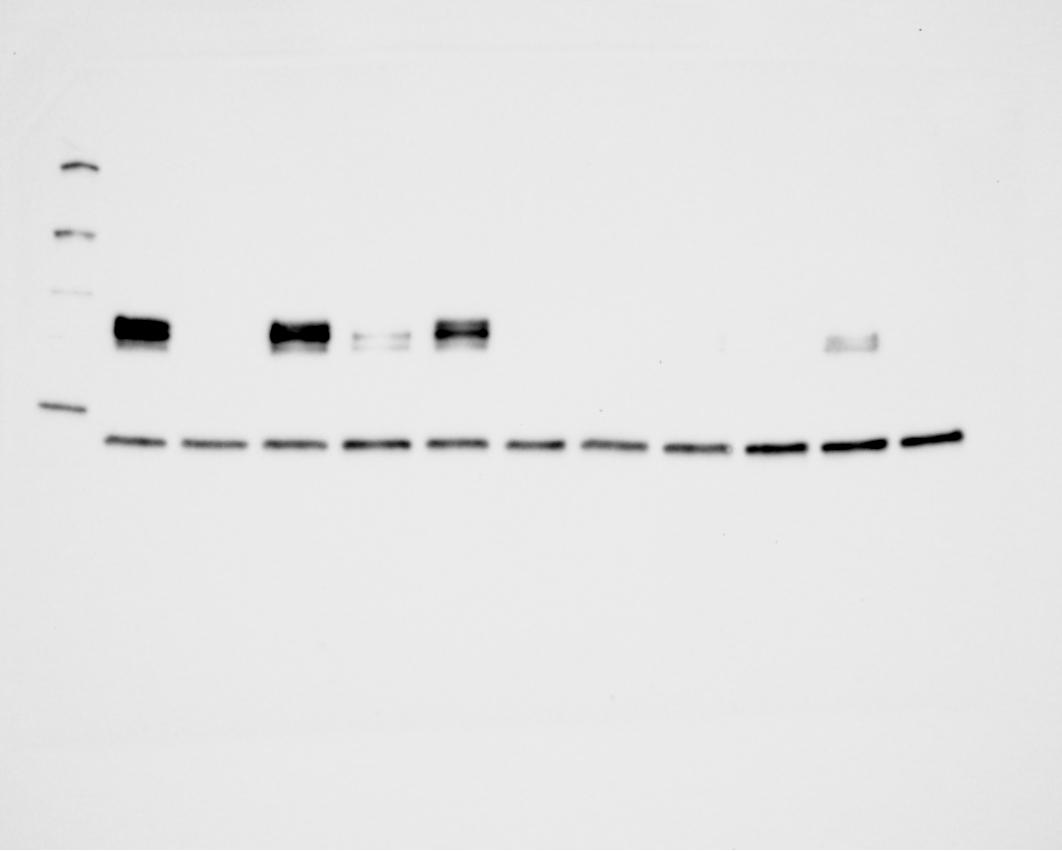

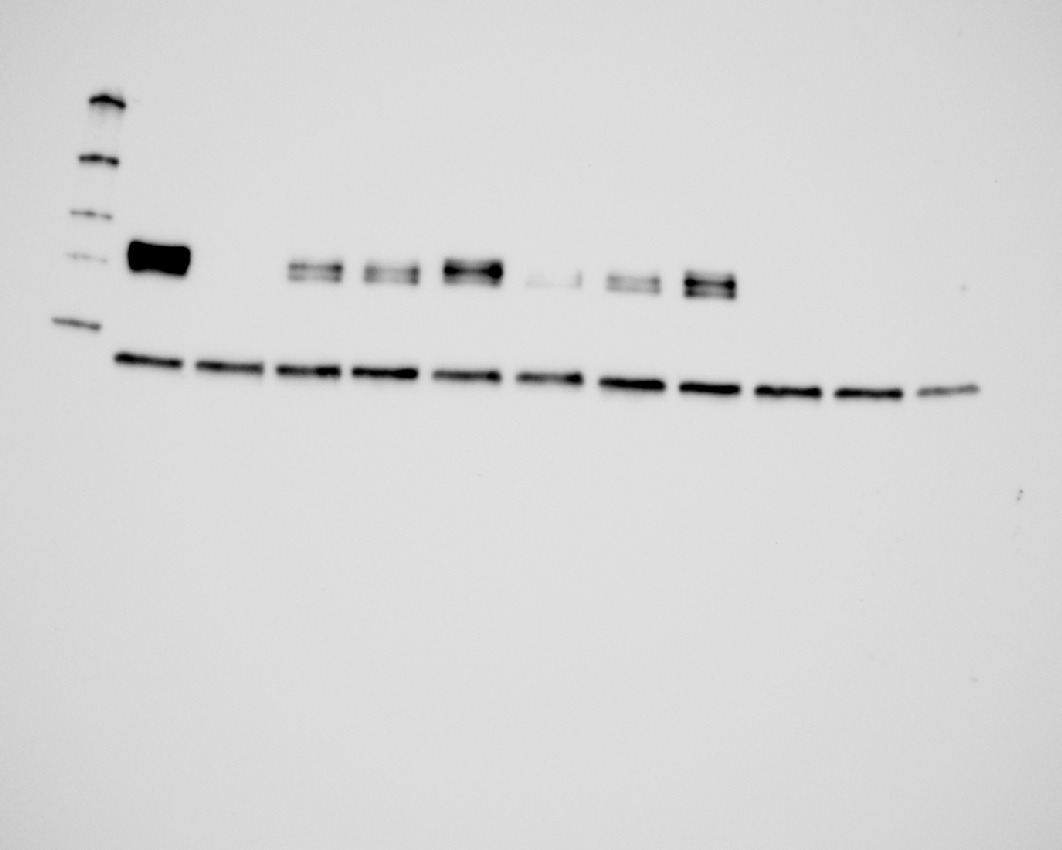


Replicate 2.


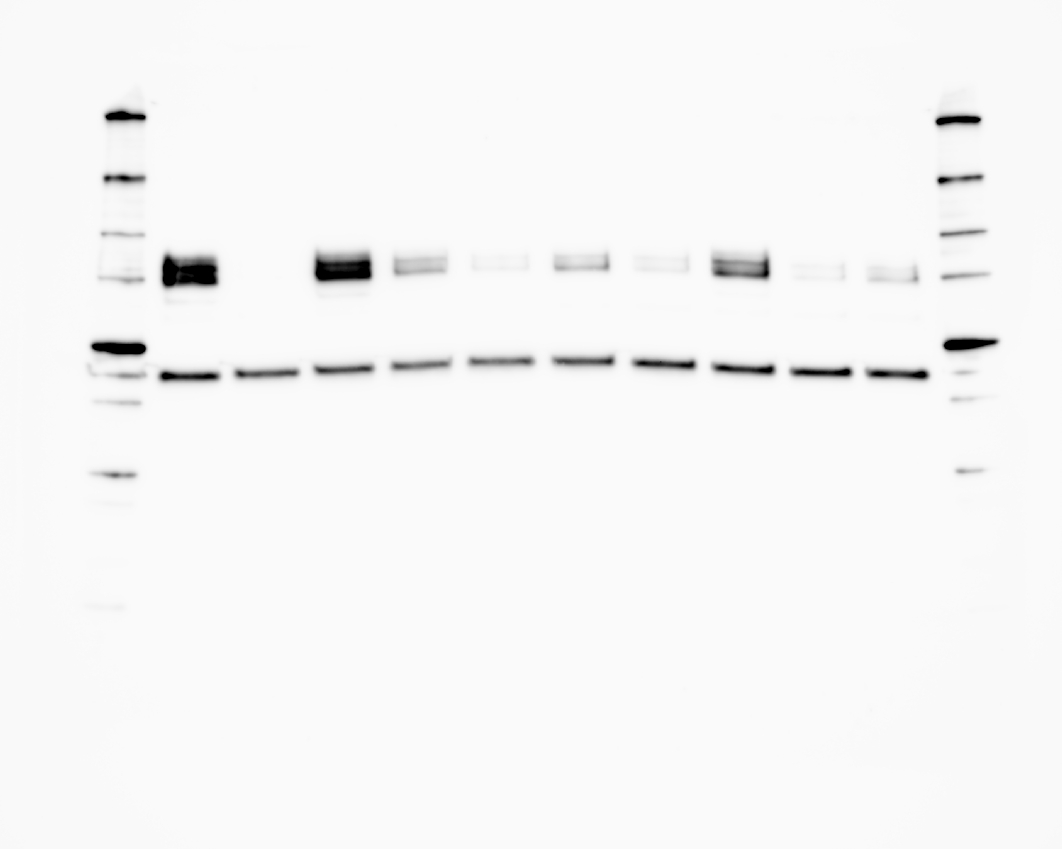

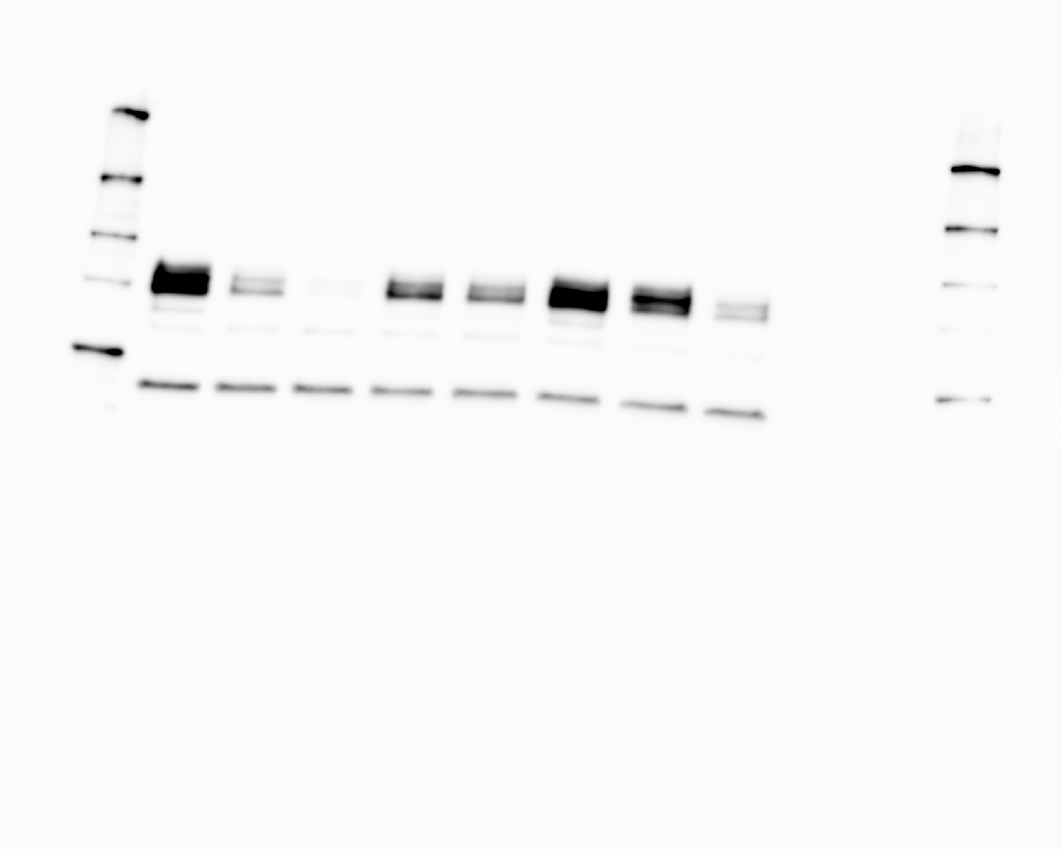

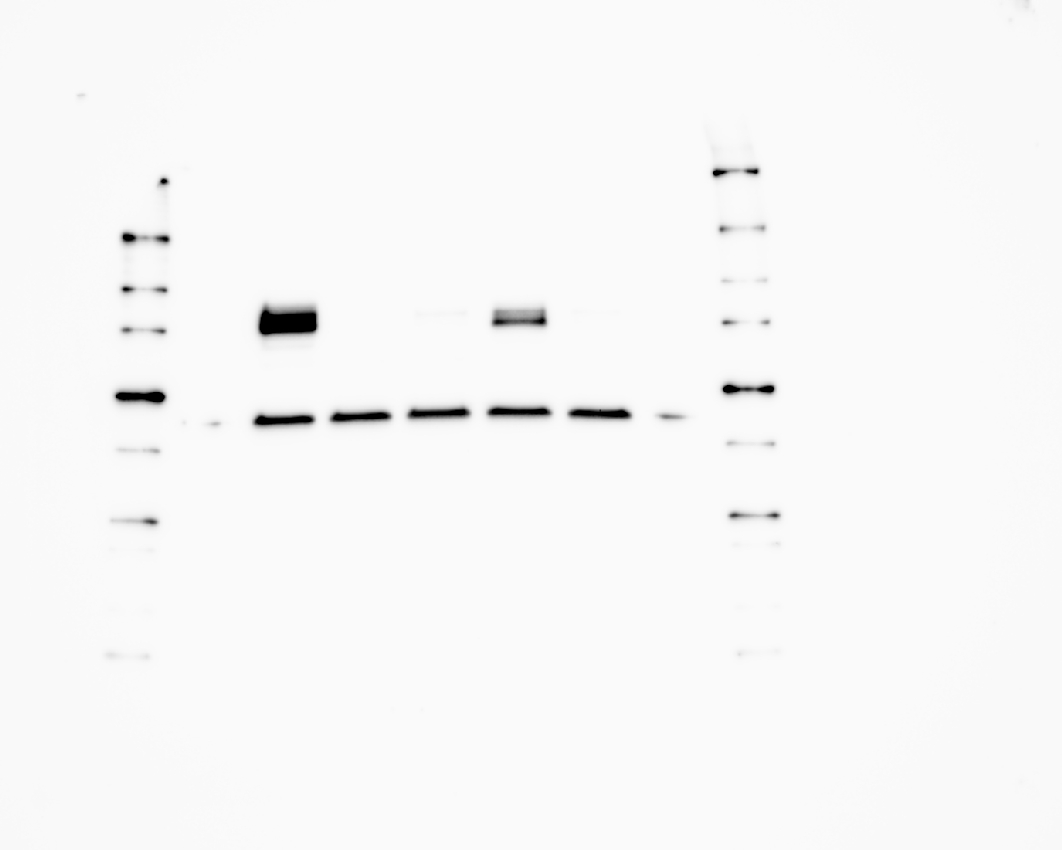


Replicate 3.


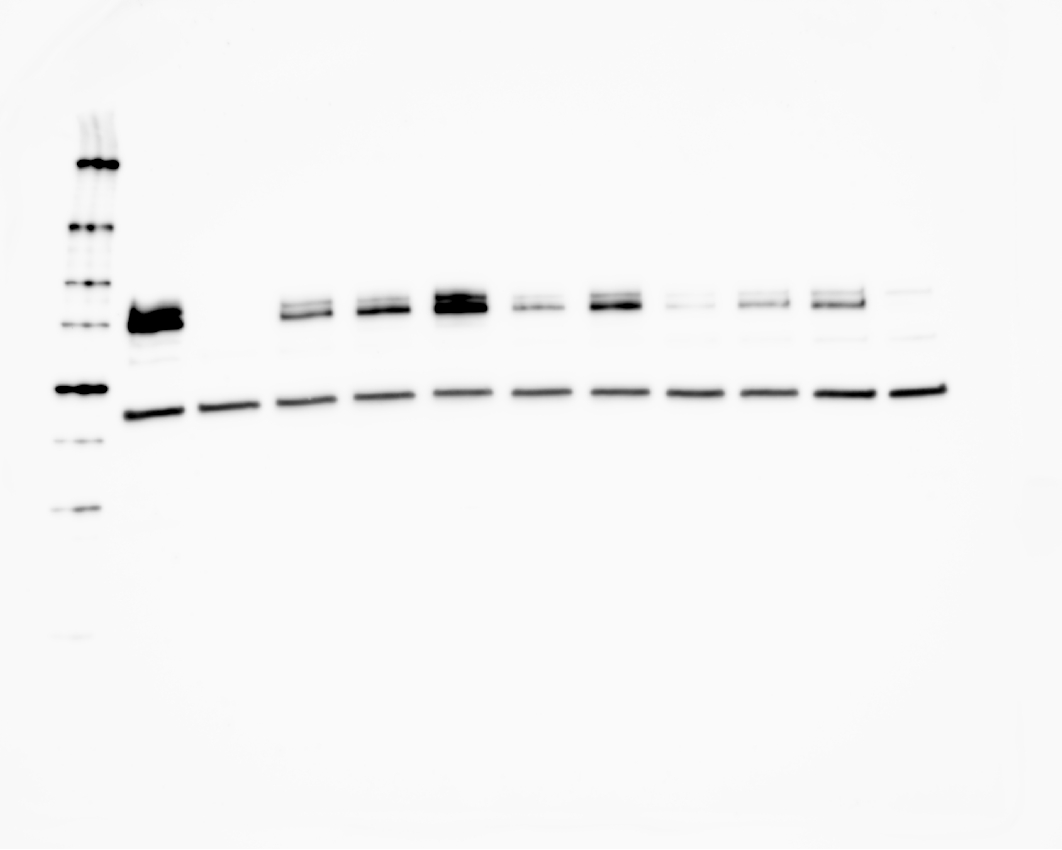

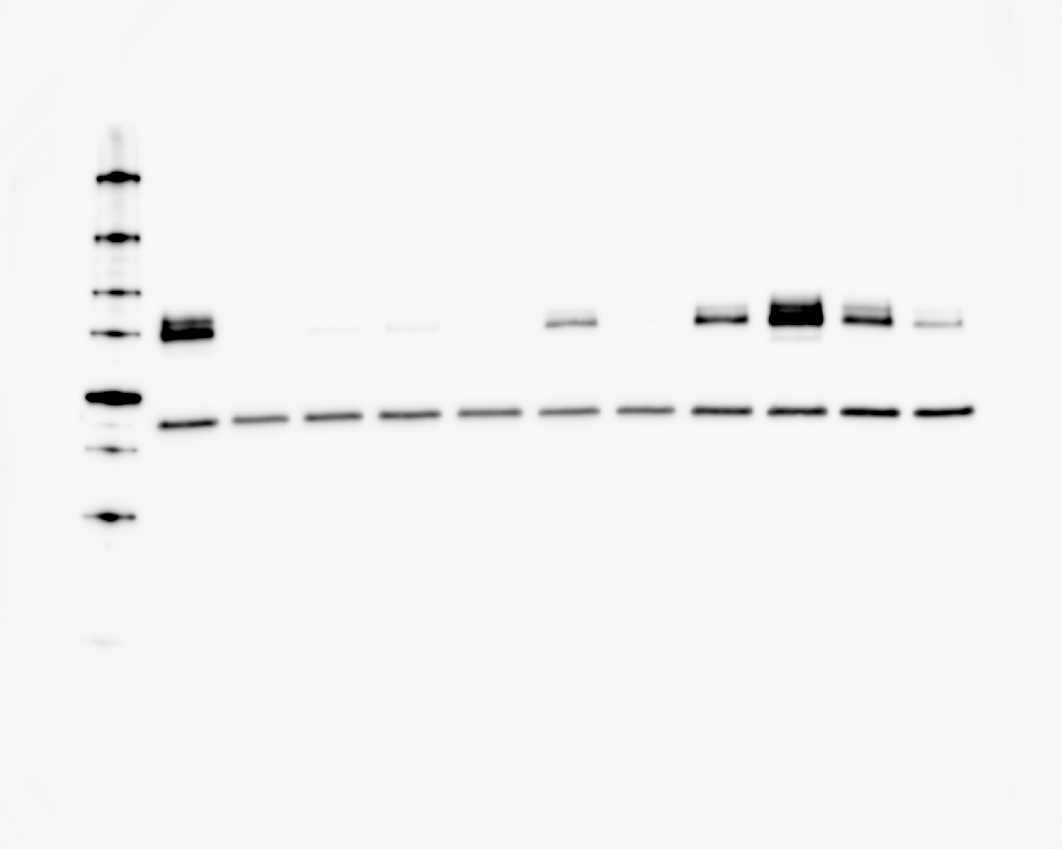


Replicate 4.


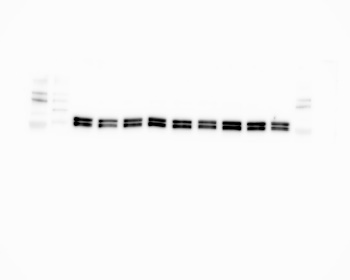


**
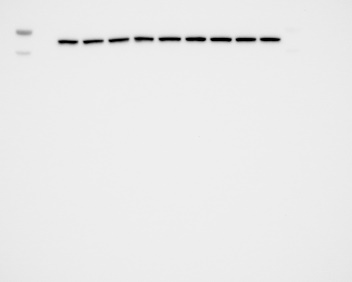
**

**B**

Replicate 1.


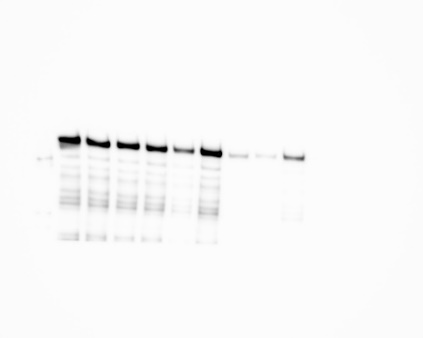
**
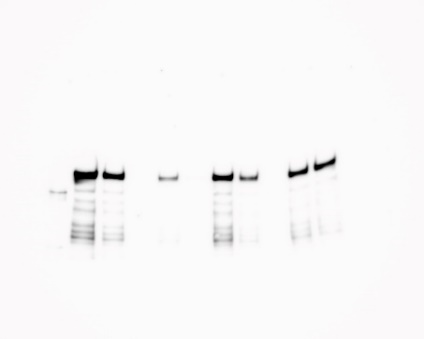
**


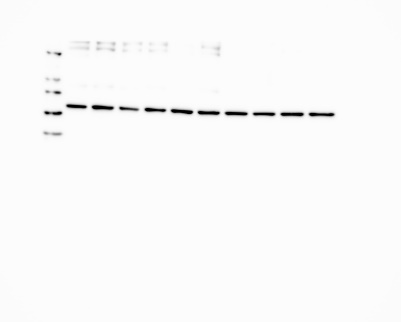
**
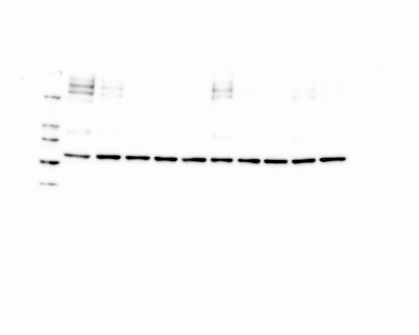
**

Replicate 2:


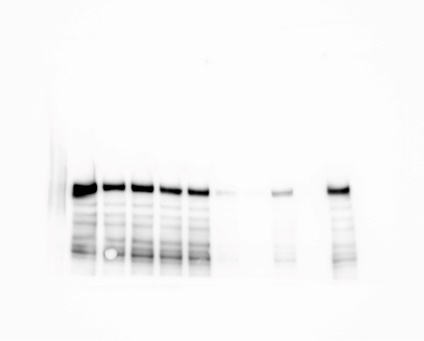

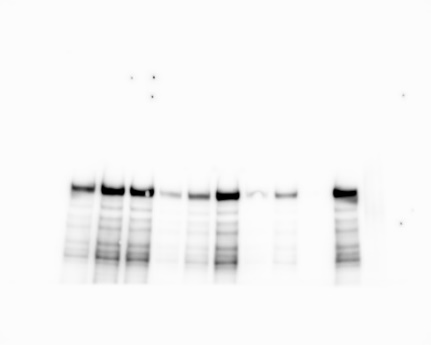


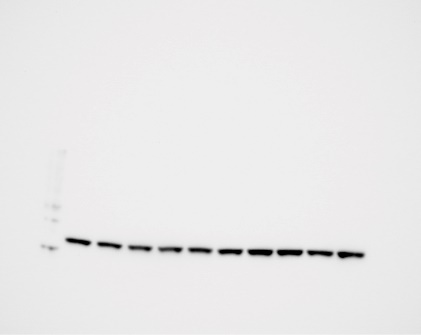

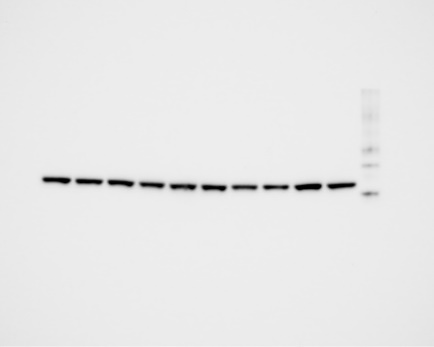


Replicate 3:

**
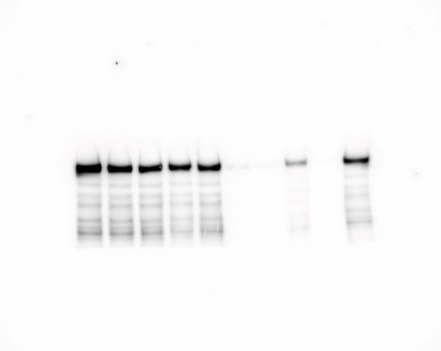
**
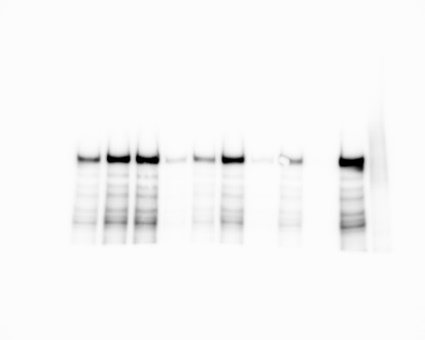


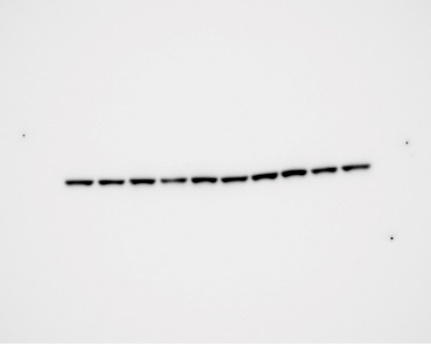

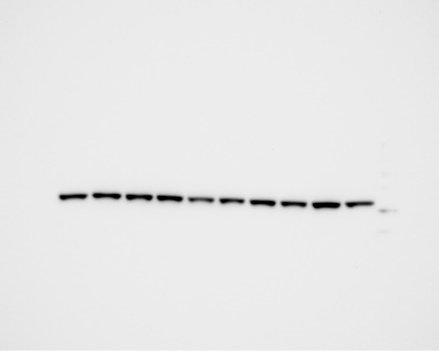


Replicate 4:


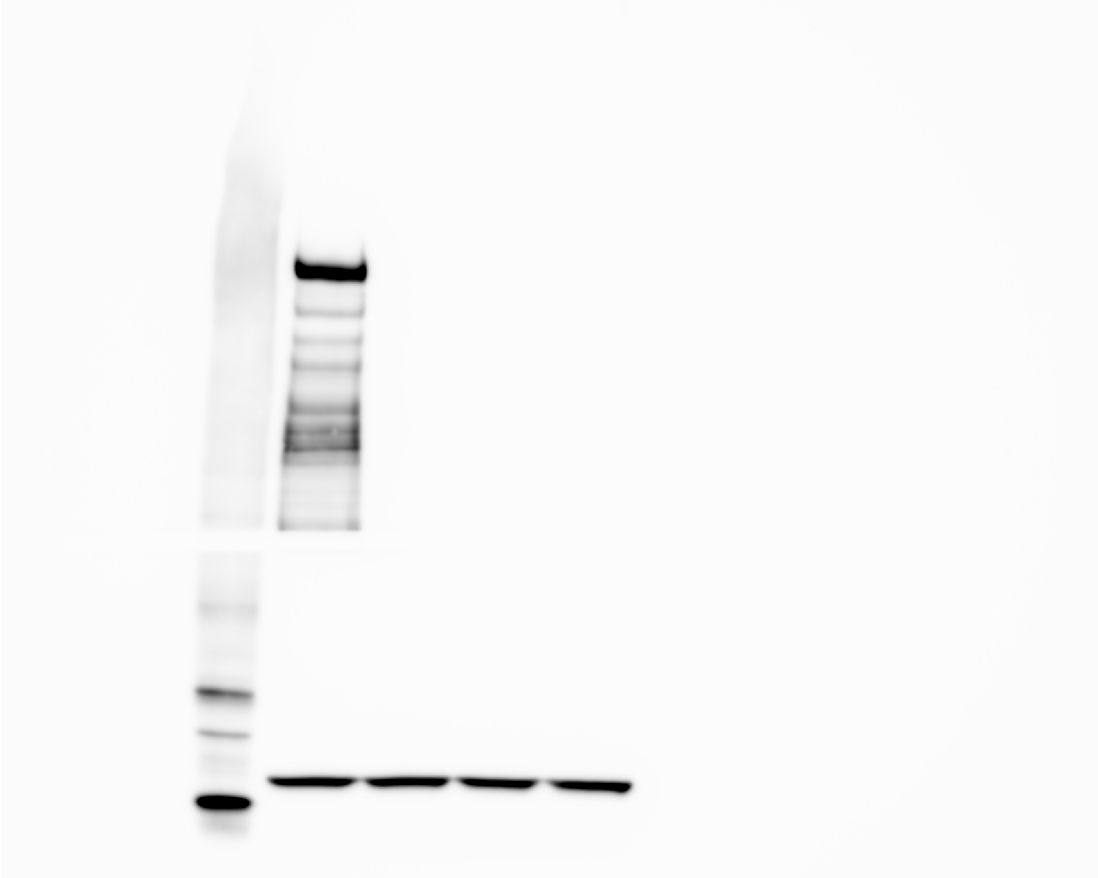


**Supplementary figure 4: Original western blot images for Supplementary figure 1.** The images are not cropped or edited. **(A)** The figure shows original images from four replicates for the fusion protein DBD-BRCT. Loading order replicate 1: WT, EV, p.Leu1439Phe, p.Phe1668Leu. p.Leu1701Met, p.Ala1708Val, p.Gly1709Arg, p.Lys1711Ser, p.Trp1718Ser, p.Pro1749Ala, not relevant, WT, EV, not relevant*, not relevant, p.Arg1835Gln, not relevant, p.Met1652Ile, p.Val1804Asp, not relevant, p.Arg1699Trp, p.Val1838Gly. Loading order replicate 2: WT, EV, p.Leu1439Phe. p.Met1652Ile, not relevant, p.Leu1701Met, p.Arg1699Trp, p.Leu1701Met, p.Ala1708Val, p.Gly1709Arg, WT, p.Lys1711Ser, not relevant, p.Val1804Asp, not relevant, not relevant, p.Arg1835Gln, not relevant, WT, EV, p.Trp1718Ser, p.Pro1749Ser, p.Val1838Gly. Loading order replicate 3: WT, EV, p.Met1652Ile, p.Val1804Asp, p.Leu1439Phe, p.Phe1668Leu, p.Leu1701Met, p.Ala1708Val, p.Gly1709Arg, p.Lys1711Ser, p.Trp1718Ser, WT, EV, p.Ser1655Phe, p.Arg1699Trp, p.Val1838Gly, p.Pro1749Ala, not relevant, not relevant, not relevant, p.Arg1835Gln, not relevant. Loading order replicate 4: WT, p.Glu1535Lys, p.Met1628Ile, WT, p.Glu1535Lys, p.Met1628Ile, WT, p.Glu1535Lys, p.Met1628Ile. BRCA1 was detected with anti-BRCA1 antibody. The BRCA1 bands were normalised against the total protein of each lane. **(B)** The figure shows images from four replicates for the full-length His-BRCA1. The blots were cut in two prior to incubation with anti-BRCA1 and anti-actin, so each blot are divided into two images. Loading order replicate 1, 2 and 3: WT, p.Leu1439Phe, p.Glu1535Lys, p.Met1628Ile, p.Phe1668Leu, p.Leu1701Met, p.Ala1708Val, p.Gly1709Arg, p.Lys1711Gn, p.Trp1718Ser, WT, not relevant, not relevant, not relevant, p.Pro1749Ala, p.Arg1835Gln, p.Arg1699Trp, p.Val1838Gly, p.Met1652Ile, p.Val1804Asp. Loading order replicate 4: WT, EV, EV, EV. BRCA1 was detected with anti-BRCA1 antibody. Actin was used as loading control to normalise the corresponding BRCA1 bands.

*Samples included in the Western Blot, but not relevant in this study.
